# The Comparative Characterization of a Hypervirulent *Acinetobacter baumannii* Bacteremia Clinical Isolate Reveals a Novel Mechanism of Pathogenesis

**DOI:** 10.3390/ijms25189780

**Published:** 2024-09-10

**Authors:** Payam Benyamini

**Affiliations:** The Lundquist Institute for Biomedical Innovation at Harbor-UCLA Medical Center, Torrance, CA 90502, USA; payamb@ucla.edu

**Keywords:** infectious diseases, *Acinetobacter baumannii*, Gram-negative bacteria, mechanism of pathogenesis, Zonula occludens toxin (Zot), biofilms, multidrug resistance, quorum sensing, stealth infection

## Abstract

*Acinetobacter baumannii* is an opportunistic Gram-negative pathogen with exquisite survival capabilities under various environmental conditions and displays widespread resistance to common antibiotics. *A. baumannii* is a leading cause of nosocomial infections that result in high morbidity and mortality rates. Accordingly, when multidrug resistance rates surpass threshold levels, the percentage of *A. baumannii* clinical isolates surges. Research into *A. baumannii* has increased in the past decade, and multiple mechanisms of pathogenesis have been identified, including mechanisms underlying biofilm development, quorum sensing, exotoxin production, secretion system utilization, and more. To date, the two gold-standard strains used to investigate different aspects of *A. baumannii* pathogenesis include ATCC 17978 and ATCC 19606. Here, we report a comparative characterization study of three additional *A. baumannii* clinical isolates obtained from different infection types and derived from different anatomical regions of infected patients. The comparison of three clinical isolates in addition to the ATCC strains revealed that the hypervirulent bacteremia clinical isolate, known as HUMC1, employs a completely different mechanism of pathogenesis when compared to all its counterparts. In stark contrast to the other genetic variants, the hypervirulent HUMC1 isolate does not form biofilms, is antibiotic-susceptible, and has the capacity to reach higher levels of quorum compared to the other clinically relevant strains. Our data also reveal that HUMC1 does not shed endotoxin into the extracellular milieu, rather secretes the evolutionarily conserved, host-mimicking, Zonula occludens toxin (Zot). Taken together, our hypothesis that HUMC1 cells have the ability to reach higher levels of quorum and lack biofilm production and endotoxin shedding, accompanied by the substantial elaboration of Zot, suggests a novel mechanism of pathogenesis that appears to afford the hypervirulent pathogen with stealth-like capabilities when disseminating through the circulatory system in a state of bacteremia.

## 1. Introduction

Drug-resistant pathogens such as Gram-negative bacteria (GNB) have emerged as a serious global threat to both civilian and military medical facilities [1,2,3,4,5,6,7,8]. *Acinetobacter baumannii* is an opportunistic GNB commonly considered a multidrug-resistant (MDR) pathogen acquired during long-term stays in clinical settings [3,9,10,11,12,13]. *A. baumannii* infections manifest as serious diseases particularly in cases of bacteremia, invasive wound infections, meningitis, pneumonia, and urinary tract infections [14,15,16,17]. Clinical studies reveal that chronic antibiotic use and mechanical ventilation are the primary causes that lead to infection by *A. baumannii* [18,19]. MDR *A. baumannii* pathogens are associated with high mortality rates up to 40–80% [20]. *A. baumannii’s* ability to persist in hospitals or long-term care facilities and rapidly acquire antibiotic resistance has made this MDR pathogen one of the predominant nosocomial infections worldwide [1,3,9,20]. The rapid rise in different MDR *A. baumannii* genetic variants is a great concern, and it is listed as a priority pathogen by the World Health Organization [21]. It is estimated that MDR *A. baumannii* strains increased from <4% in 2000 to over 60–70% by 2010 [1,9,10,22,23]. These newly emerging *A. baumannii* genetic variants are becoming more prevalent and continue to proliferate due to the lack of novel antimicrobials in the drug development pipeline [1,21,22,23,24,25,26,27,28,29]. MDR *A. baumannii* infections are associated with greater health care costs, longer hospital stays, greater morbidity, and >60% mortality for bacteremia cases as compared to drug-susceptible strains [29].

The invasive mechanisms used by *A. baumannii* rely on its ability to produce virulence factors such as biofilms, secretion systems, quorum sensing, endotoxin shedding, and the elaboration of exotoxins [30,31]. These factors are employed or produced depending on where and when the bacterium establishes itself within the host. Unfortunately, knowledge surrounding alternative mechanisms of pathogenesis among different virulent *A. baumannii* clinical isolates has been historically limited. Here, we report the comparative characterization of five different clinically relevant *A. baumannii* isolates derived from different anatomical regions: HUMC1 (bacteremia), HUMC6 (ventilator-associated pneumonia), HUMC12 (diabetic stump wound), ATCC 17978 (fetal meningitis), and ATCC 19606 (urinary tract) [32,33,34]. Our data reveal the identification of a novel mechanism of pathogenesis employed by the hypervirulent bacteremia strain known as HUMC1. Its pathogenesis is completely different from the rest of the clinical strains tested. In stark contrast to the other isolates, HUMC1 reaches significantly higher levels of quorum under all conditions tested, is not extensively MDR, does not form biofilms, does not shed endotoxin, and utilizes fluctuations in cell density to regulate the secretion of high concentrations of Zonula occludens toxin (Zot) into the extracellular milieu.

## 2. Results

### 2.1. Different Acinetobacter Baumannii Clinical Isolates Responded Differently to Antibiotic Treatment

**Figure 1:**
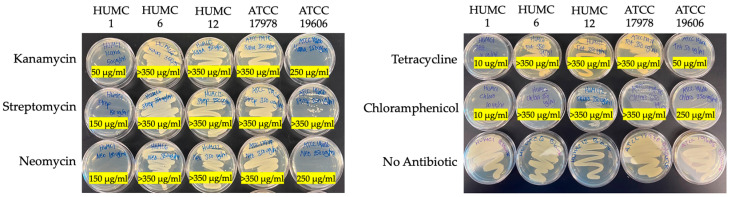
Minimum inhibitory concentration experiments, in the context of surface growth, reveal varying levels of resistance among different clinical isolates. HUMC12 is the most MDR, followed by HUMC6 and ATCC 17978. Strain ATCC 19606 is susceptible to high concentrations of antibiotics. HUMC1 is the most sensitive to moderate levels of antibiotics, compared to its counterparts.

We performed minimum inhibitory concentration (MIC) assays, in the context of surface growth, to assess the antibiotic susceptibility profile of the five different *A. baumannii* stains. Our data show clinical isolate HUMC12 to be the most drug resistant of all isolates, including resistance to chloramphenicol (>350 µg/mL). HUMC6 and ATCC 17978 are also extensively resistant to most different antibiotics tested (>350 µg/mL), except for chloramphenicol. ATCC 19606 is susceptible to high concentrations of antibiotics ranging between 50 and 350 µg/mL. The HUMC1 clinical isolate is most susceptible to moderate concentrations of antibiotics ranging between 10 and 150 µg/mL (Figure 1). It should be noted that antibiotic resistance profiles vary when comparing different MICs in cell suspensions versus association with surface growth, except for HUMC1 which is similar [35,36,37].

### 2.2. Hypervirulent HUMC1 Clinical Isolate Does Not Form a Biofilm

**Figure 2:**
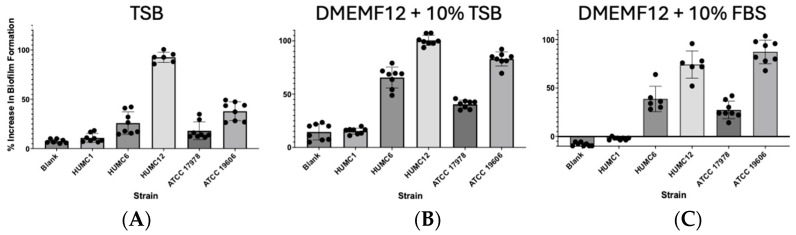
Biofilm formation of tested isolates reveals that the hypervirulent clinical isolate HUMC1 does not develop a biofilm. Biofilm production was evaluated under different media conditions: 100% TSB (**A**); DMEMF12/10% TSB (**B**); DMEMF12/10% FBS (**C**). Tryptic soy broth (TSB); fetal bovine serum (FBS).

Biofilms are communities of microorganisms encased in an exopolysaccharide substance (EPS). Biofilms are defined by an assemblage of cells tightly adherent onto inert surfaces or living cells [38]. Biofilms protect the bacterium from high-dose antibiotic treatments and phagocytosis by cells of the immune system [39,40]. Our studies show that the clinically relevant isolates HUMC12 and ATCC 19606 produced the highest level of biofilm development when cultured under all growth conditions. In comparison, HUMC6 and ATCC 17978 produced moderate levels of biofilm. Surprisingly, the hypervirulent HUMC1 clinical isolate did not show any adhesive or biofilm production capabilities when cultured in different media conditions (Figure 2). The comparison between antibiotic resistance profiles, presented in Figure 1, and the biofilm data displayed in Figure 2 revealed a correlation between the susceptibility of the different strains to a panel of antibiotics and the levels of biofilm production. For example, the hypervirulent HUMC1 clinical isolate did not form any biofilms and therefore was the most susceptible to antibiotic treatment. In contrast, the HUMC12 isolate produced the highest level of biofilm and was also the most drug resistant. Clinical isolates HUMC6 and ATCC 17978 produced moderate levels of biofilm and were resistant to most antibiotics tested, except for chloramphenicol. Interestingly, clinical isolate ATCC 19606 formed extensive biofilms and was resistant to most antibiotics but not all. 

### 2.3. The Hypervirulent HUMC1 Clinical Isolate Reached Twice the Cell Density When Compared to Its Counterparts

**Figure 3:**
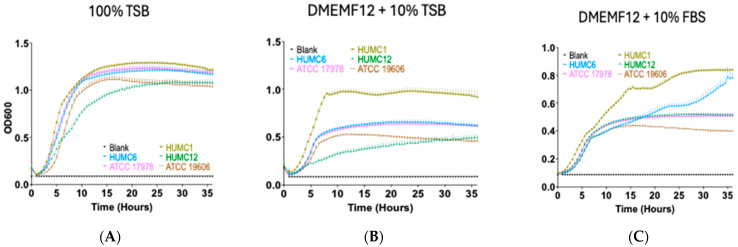
Growth kinetics of hypervirulent HUMC1 cells show twice the cell density compared to other clinical isolates. Growth kinetics (using OD @ 600 nm) of HUMC1, HUMC6, and ATCC 17978 cultured for 36 h, using TSB only (**A**), DMEMF12 + 10% TSB (**B**), and DMEMF12 + 10% FBS to mimic human physiological conditions (**C**). Note the increased cell density achieved by HUMC1 cells in all media, compared to HUMC6, HUMC12, ATCC 17978, or ATCC 19606. HUMC6 reaches similar cell density only when cultured in DMEMF12 supplemented with 10% FBS.

Virulence factor production is a highly regulated process and usually occurs based on local cell density [41,42]. The stationary phase is the stage when growth ceases but cells remain metabolically active [43,44]. During this phase, a wide variety of secondary metabolites, antibiotics, and toxins are synthesized [44,45]. Thus far, it is not well known which selective pressures shape the *A. baumannii* evolution of cell-density-dependent growth. Accordingly, we assessed strain-specific growth kinetics, using three different types of culture media, including media that recapitulate physiological conditions (DMEMF12 supplemented with 10% fetal bovine serum (FBS) or 10% tryptic soy broth (TSB)) vs. growth in enriched bacterial culture media such as 100% TSB. Our kinetic studies showed that when cultured in enriched TSB media, all strains replicated at approximately the same rate and reached similar levels of cell density (Figure 3A). In stark contrast, when grown in culture media such as DMEMF12 supplemented with 10% TSB or 10% FBS, hypervirulent HUMC1 cells displayed extended replicative capacity and reached twice the cell density compared to the rest of the clinical isolates (Figure 3B), except for HUMC6, which reached similar levels of cell density to those of HUMC1 cells when grown under physiological conditions but at a much later timepoint during growth (Figure 3C). HUMC6 reached higher levels of quorum when cultured in DMEMF12 + 10% FBS (Figure 3C). In all cases, our kinetic data revealed that hypervirulent HUMC1 cells reached twice the cell density irrespective of growth conditions (Figure 3). Interestingly, the HUMC1 strains’ extended replicative capacity explains their inability to form biofilms and their higher susceptibility to antibiotic treatment, specifically to antibiotics that target growth-related functions. Our studies also show a correlation between cell density and biofilm formation. HUMC12 and ATCC 19606 cells showed the highest level of biofilm production, and their capacity to increase cell density was the least among the group (Figure 2 and Figure 3).

### 2.4. The Hypervirulent HUMC1 Clinical Isolate Does Not Shed Endotoxin into the Extracellular Milieu

Gram-negative cell wall components such as endotoxin are well-known potent inflammatory agents [46]. Their continued shedding from bacteria provides an easy explanation for their contribution to sepsis [47]. *A. baumannii* being a Gram-negative bacterium suggests that endotoxin shedding might be a mechanism associated with its pathogenesis. Interestingly, using endotoxin quantification methods, we show that the hypervirulent HUMC1 strain did not shed endotoxin under all conditions tested. In contrast, its counterparts were all endotoxin shedders, irrespective of culture media (Table 1). Strains HUMC6, HUMC12, ATCC 17978, and ATCC 19606 were all extensive shedders when cultured in TSB, followed by being strong shedders in media containing DMEMF12 supplemented with 10% TSB or 10% FBS. It should be noted that HUMC1 is a bacteremia clinical isolate in a dissemination phase of infection, while all the other strains form biofilms and cause local infections. This suggests that when HUMC1 is disseminating through the circulatory system it does not shed endotoxin, rather reaches higher levels of cell density and travels unnoticed by avoiding the induction of septic shock. The lack of endotoxin shedding affords HUMC1 stealth-like dissemination capabilities in the circulatory system.

### 2.5. The Hypervirulent HUMC1 Clinical Isolate Secretes an Exotoxin into the Extracellular Milieu

Our cell morphology studies revealed that when alveolar epithelial cells (A549) were incubated with cell-free supernatants from HUMC1 cultures, they detached from the culture plate and rapidly adopted a round morphology. Additionally, we identified that the substance inducing this detached and rounded state was heat-sensitive, strongly suggesting that the observed change in cell morphology is due to a secreted protein molecule. To confirm the proteinaceous nature of this substance, microscopy in conjunction with heat inactivation experiments were performed using cell-free culture supernatants.

**Figure 4:**
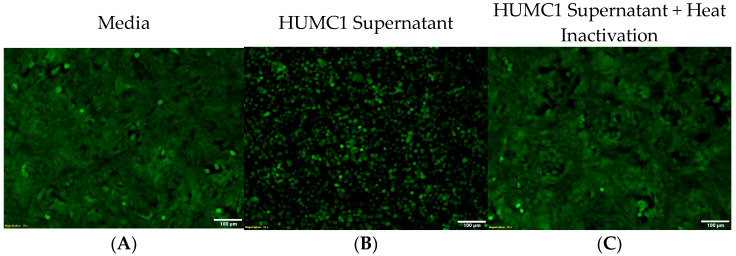
Hypervirulent HUMC1 strain elaborates a proteinaceous toxin. Microscopy images showing A549 cells incubated overnight in plain media (**A**), HUMC1 cell-free culture supernatant (**B**), and heat-inactivated HUMC1 cell-free culture supernatant (**C**). [Scale bar: 100 μm].

Microscopy analysis of A549 cells revealed the formation of an intact monolayer when incubated in normal DMEMF12 culture media supplemented with 10% FBS (Figure 4A). However, the overnight incubation of A549 cells with HUMC1 cell-free culture supernatant revealed a detached and rounded cell morphology (Figure 4B). Furthermore, when the same cell-free culture supernatant was heat-treated prior to incubation with A549 cells, the morphology remained as an intact monolayer (Figure 4C), strongly suggesting that a protein product is responsible for the change in cell morphology.

### 2.6. The Hypervirulent HUMC1 Clinical Isolate Secretes Zonula Occludens Toxin (Zot) into the Extracellular Milieu

**Figure 5:**
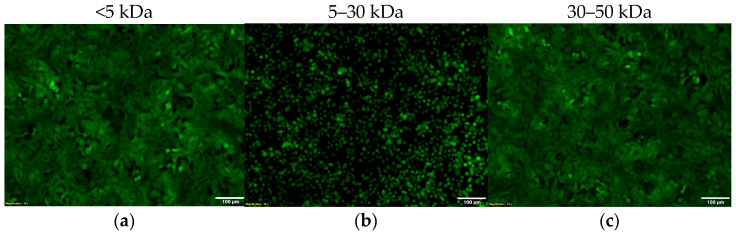
An active protein is localized to the 5–30 kDa range. Supernatant fractionations reveal an intact A549 cell monolayer when treated with a supernatant fraction below 5 kDa (**a**). A549 cells treated with fractions between 5 and 30 kDa exhibit a detached and round morphology (**b**). Incubation of A549 cells with supernatant fraction between 30 and 50 kDa shows an intact cell monolayer (**c**). [Scale bar: 100 μm].

To date, only one study has shown that numerous Gram-negative bacteria (GNB), including *A. baumannii*, secrete an evolutionarily conserved, host-mimicking toxin capable of disassembling tight junctions between adjacent epithelial cells [31]. Our fractionation studies showed that the active component responsible for the change observed in A549 cell morphology is in a molecular mass range of between 5 and 30 kDa (Figure 5). A549 cells incubated with fractions <5 kDa and between 30 and 50 kDa displayed an intact monolayer. The production of this factor correlates with the molecular weight range of the evolutionarily conserved Zonula occludens toxin (Zot) [31,48]. It is well established that numerous GNB horizontally acquired the Zot encoding gene, including *A. baumannii* [31]. While the predicted Zot molecular weight is 44.8 kDa, published reports reveal that the active component is localized to fractionations between 10 and 30 kDa [48]. This is the same molecular weight range as that of our detected exotoxin. 

### 2.7. The Activity of Zot Is Reversible

**Figure 6:**
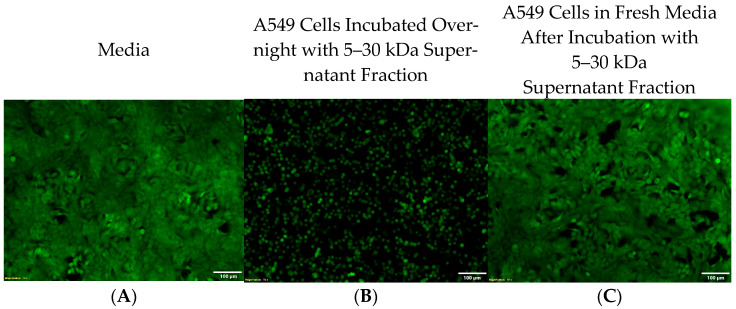
Cell detachment is reversible. A549 cells incubated in fresh media (**A**)**.** A549 cells incubated with 5–30 kDa fraction reveal a detached and rounded cell morphology (**B**). Overnight incubation of detached A549 cells in fresh media re-establishes an intact monolayer (**C**). [Scale bar: 100 μm].

The active component of Zot functions by inducing the disassembly of tight junctions (TJs) between adjacent epithelial cells resulting in a detached and rounded cell morphology [49,50]. Zot’s TJ regulatory functions are reversible and time- and dose-dependent [50]. Interestingly, these characteristics are consistent with the morphology and functional reversibility we observe after incubating A549 cells with HUMC1 culture supernatant fractions between 5 and 30 kDa, where A549 cells appear round and detached from the culture plate (Figure 6A,B). To determine the reversibility of this effect, detached cells were collected, washed, and re-incubated overnight in fresh media. Our microscopy data revealed that the detached A549 cells re-established an intact monolayer when re-incubated in fresh media, similar to the monolayer observed prior to incubation with fractionated HUMC1 cell-free culture supernatant (Figure 6C). 

### 2.8. Phylogenetic Analysis of Numerous A. baumannii Genomes Reveals All Strains Tested Harbor the Gene Encoding Zonula Occludens Toxin

**Figure 7:**
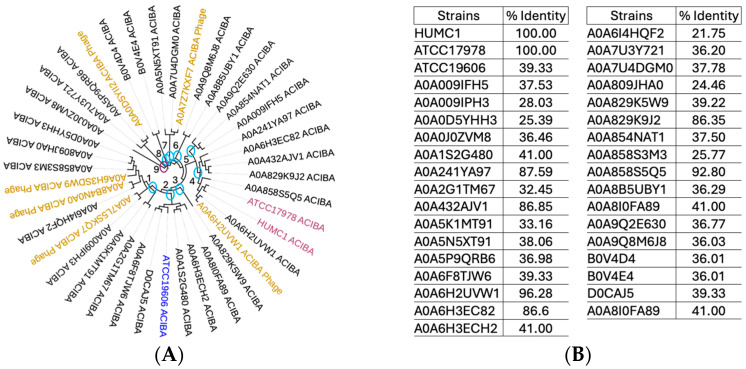
Phylogenetics of *A. baumannii (AB)*-encoded Zot reveals a high degree of sequence heterogeneity between different strains. A phylogenetic tree shows the evolutionary relatedness of Zot protein encoded among 35 different *AB* strains (**A**). Percent identity of amino acid sequences compared among 35 different *AB* strains (**B**). Notice the high degree of evolutionary relatedness and percent identity between HUMC1 and ATCC 17978.

Zot exhibits properties that enhance the permeation between epithelial monolayers [49,50]. It is an outer membrane protein originally discovered in toxigenic *Vibrio cholerae* [48]. The toxin’s horizontal passage among so many different GNB pathogens stresses its evolutionary importance [31]. Zot’s primary function is to act as a ligand that transmits a signaling cascade leading to the TJ disassembly of epithelial monolayers [49]. To determine the phylogenetic relationship between the Zot protein encoded by the HUMC1 clinical isolate to other *A. baumannii* genetic variants, in silico techniques such as proteomic-based phylogenetic tracing were employed. Our studies revealed a wide range of Zot amino acid sequence identities that are shared among 35 different *A. baumannii* strains tested. Six different bacteriophages were also included to assess the horizontal gene transfer of Zot among the different strains analyzed (Figure 7A). Our data revealed a percent identity range between 21 and 100% (Figure 7B). Phylogenetic analysis of all different *A. baumannii* strains revealed them to be distributed among nine clades and two outgroup lineages. Clade one is made of five clusters. Clade two comprises four clusters, which also include the clinical isolate ATCC 19606 as one of the strains. Clade three comprises a single *A. baumannii* genetic variant. Clade four has only three clusters, including HUMC1 and ATCC 17978 strains in the same cluster. Interestingly, clinical isolates HUMC1 and ATCC 17978 share 98% genome homology but thus far have displayed completely opposite phenotypic characteristics. Their Zot-encoded amino acid sequences are 100% identical. Clade five is made of two clusters. Clades six, seven, and eight comprise a single cluster each. Lastly, two outgroup lineages have also been identified, both of which share minimal ancestry to the other clades. The assessment of a horizontal passage of Zot genes among all strains revealed three phages present in clade one. A single phage was found in the same clade containing clinical isolates HUMC1 and ATCC 17978. Clades six and eight each had one phage present.

### 2.9. Protein Sequence Alignments and Structural Modeling of Zot Encoded by V. cholerae vs. A. baumannii Show a Low Degree of Amino Acid Sequence Identity with a High Degree of Structural Similarity

**Figure 8:**
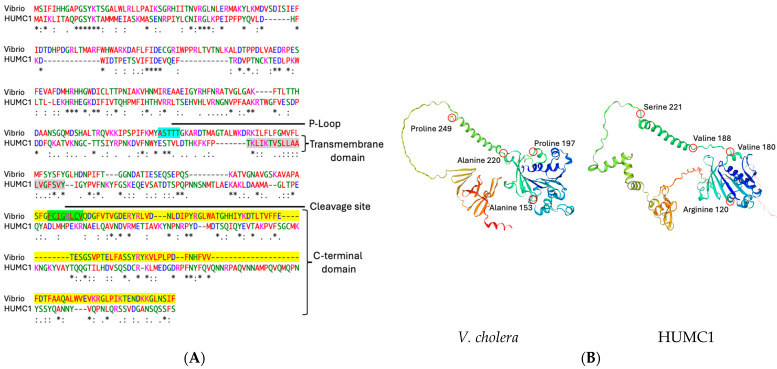
Comparative sequence analysis of Zot encoded by *V. cholerae* vs. HUMC1 reveals the presence of multiple functional domains. Amino acid sequence comparisons between the two pathogens reveal the presence of a Walker motif, a transmembrane domain, a cleavage site, and a carboxy terminal domain (**A**). Zot protein encoded by both pathogens reveals a very high degree of structural homology (**B**). [Green, polar uncharged; magenta, positively charged; red, hydrophobic; blue, negatively charged]; [Conserved identical sequence (*); conserved mutation (:); semiconservative mutation (.)].

Zot is associated with the outer membrane of *V. cholerae* and has a molecular mass of approximately 45 kDa [48,50]. This transmembrane protein is proteolytically processed at a specific cleavage site present on its carboxy terminus [31,50]. The final toxigenic fragment is released into the extracellular milieu, where it binds specific receptors on epithelial cells and initiates the disassembly of TJs [49]. Since Zot was originally discovered in *V. cholerae*, the proteomic comparison of Zot amino acid sequences derived from HUMC1 vs. *V. cholerae* revealed 23% identity among the two pathogens (Figure 8). Our comparisons show the presence of a conserved P-loop domain, suggesting ATPase activity. A transmembrane domain has been identified as well. Our comparisons also show that the carboxy terminal cleavage site for the *V. cholerae*-specific Zot protein has a completely different amino acid sequence from Zot encoded by HUMC1, suggesting niche-specific alternative processing at the carboxyl terminus. 

Comparative structural modeling reveals that even though there is very low sequence identity between HUMC1 and *V. cholerae*, structurally they exhibit a very high degree of structural homology, specifically amino acids spanning from alanine 153 to proline 249 in *V. cholera* and amino acids between arginine 120 and serine 221 in HUMC1. Both stretches start with the formation of a β-sheet, comprising three β-strands, followed by a long α-helical conformation. The remaining amino acids after proline 249 in *V. cholerae* and serine 221 in HUMC1 reveal the presence of an additional domain that adopts a completely different structural conformation when comparing the two pathogens. The additional domain of *V. cholerae*-encoded Zot protein is organized into several β-sheets and an α-helix at the amino terminus. The second domain of HUMC1-encoded Zot protein adopts several α-helical structures, without any β-strands, strongly suggesting the presence of niche-specific structural functions.

### 2.10. The Hypervirulent HUMC1 Clinical Isolate Is Fitter than Its Counterparts

**Figure 9:**
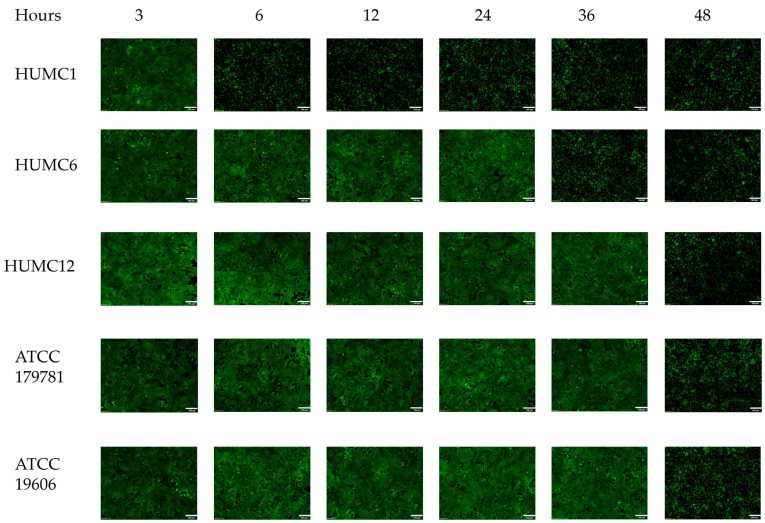
Hypervirulent HUMC1 clinical isolate secretes Zot at earlier timepoints during growth. Time-course experiments using cell-free supernatants collected from all strains at different time intervals show that the onset and extent of Zot secretion is associated with an ability to reach increased levels of cell density at approximately 6 h growth. Note the significant increase in A549 cell detachment at 6 h for the hypervirulent HUMC1 strain, compared to its counterparts. Culture supernatants from all strains, containing DMEMF12 media supplemented with 10% FBS, were used to culture the A549 cells. [Scale bar: 100 μm].

Different *A. baumannii* strains can cause infection in mice and can produce Zot with activity against A549 alveolar epithelial cells, suggesting that either the toxin has little contribution to virulence or there is a difference in fitness among the strains that leads to altered toxin concentration. The latter assumption is supported by both our growth kinetic studies displayed in Figure 3 and time-course microscopy experiments using A549 cells incubated with cell-fee culture supernatants, collected at different timepoints during growth, shown in Figure 9. Our data revealed that around the same time that HUMC1 cells begin to reach higher levels of quorum, A549 cells began to display a detached and rounded morphology (Figure 9).

We compared our growth kinetics among all five strains to establish a correlation between growth fitness (i.e., cell density) and its contribution to the secretion of Zot (Figure 3). We compared their growth with the ability of cell-free conditioned media (collected at different time intervals during growth) to cause the detachment and cell rounding of A549 cells (Figure 9). Growth curves of the bacterial isolates were assessed using DMEMF12 media supplemented with 10% FBS (mimicking host cell environment) (Figure 3C). Our data show that HUMC1 cells had twice the cell density starting at approximately seven to ten hours of growth and until the termination of the experiment at 36 h (Figure 3B,C). All the other strains remained at lower levels of cell density and replicated poorly in DMEMF12 media. Interestingly, when grown in host environmental conditions, our results revealed that the HUMC6 strain also reached higher levels of quorum during later stages of growth (Figure 3C). Accordingly, our microscopy data revealed that the onset of Zot secretion correlated with extended replicative capacity and the ability to reach higher levels of cell density (Figure 9). A549 cells incubated with HUMC1 conditioned media showed a detached and rounded morphology around the same timepoints where cell density doubled. Similarly, A549 cells incubated with supernatants derived from HUMC6 cultures also displayed a detached and rounded morphology around the same timepoint, reaching twice the cell density (Figure 9).

The superiority of the HUMC1 cells’ extended growth capabilities strongly suggests that the enhanced ability of cell-free supernatants, collected from the hypervirulent HUMC1 strain, is due to their ability to reach higher levels of quorum than the rest of the *A. baumannii* strains, translating into higher concentrations of Zot and the increased ability of cell-free culture supernatants to cause the detachment of A549 cells at a significantly earlier timepoint. Cell-free supernatants collected from HUMC12, ATCC 17978, or ATCC 19606 strains all secreted Zot but at much later time intervals, closer to 48 h.

## 3. Discussion

*Acinetobacter baumannii* is an opportunistic Gram-negative bacterium that displays outstanding survival mechanisms when interacting with a host. This pathogen’s genome harbors many genes that confer resistance to a plethora of antibiotics and encodes for virulence factors responsible for adaptation. Most *A. baumannii* infections are acquired in healthcare settings, particularly in patients who are on chronic antibiotics, are immunocompromised, or are receiving intensive care [14,51]. The most common clinical manifestations are associated with mechanical ventilation, catheters, central lines, and urinary tract, lung, and meningitis infections [9,10]. The lack of efficacious drugs has resulted in high mortality rates ranging between 40 and 80% for infections occurring in sterile sites [2,4,12]. In recent years, many *A. baumannii* isolates have become highly resistant to all antibiotics, resulting in a >15-fold increase since 2000 [1,3,9,10,13,52]. Unfortunately, new multidrug-resistant (MDR) *A. baumannii* isolates are becoming more prevalent and continue to proliferate due to the absence of new antimicrobials in the drug development pipeline [25,27,28].

In recent decades, many research efforts concerning *A. baumannii* have focused on identifying antibiotic-independent modalities to treat this devastating infectious disease [53,54,55,56,57,58]. However, efforts to identify novel virulence factors, clarifying host–pathogen interactions and identifying novel mechanisms of pathogenesis, have been lacking. The secretion of toxins is well established as being vital to the infectious processes of many bacterial pathogens [59,60,61,62,63]. Toxins are potent virulence factors produced by a wide variety of bacteria that target host cells and play an essential role in host–pathogen interactions [60,63,64,65,66]. In many cases, toxin production determines the outcome of disease [67,68,69,70]. For example, it is well established that the injection of small amounts of purified toxins from different bacterial species can recapitulate many key symptoms of disease [67,68,71,72]. Here, for the first time, we report the characterization of an *A. baumannii* bacteremia clinical isolate (HUMC1) with hypervirulent capabilities that uses an alternative mechanism of pathogenesis, compared to its counterparts. In stark contrast to the other clinical isolates tested, hypervirulent HUMC1 cells do not shed endotoxin, do not form biofilms, and are susceptible to antibiotics at lower doses. HUMC1 cells have the capability to reach significantly higher levels of quorum, while secreting extensive amounts of the evolutionarily conserved host-mimicking toxin Zonula occludens toxin (Zot). Zot is an outer membrane protein secreted into the extracellular milieu by numerous Gram-negative bacterial pathogens, including *A. baumannii* [31]. In the context of host–pathogen interaction, Zot serves as a host-mimicking protein capable of increasing epithelial cell permeability and enabling the paracellular transport of *A. baumannii* into deeper layers of epithelial tissue. The reversible nature of Zot’s mechanism of action suggest that the toxin avoids causing tissue damage and inducing a subsequent inflammatory response [49,50].

Using a variety of comparative studies between five different *A. baumannii* clinical isolates, we showed that the hypervirulent HUMC1 clinical isolate employs a completely different mechanism of pathogenesis compared to the rest of the *A. baumannii* strains tested. Except for HUMC1, all other isolates were obtained from infections associated with direct physical contact with epithelial tissue. For example, the HUMC6 strain was isolated from ventilator-associated pneumonia, HUMC12 cells were derived from a diabetic stump wound, ATCC 17978 cells were obtained from fetal meningitis, and lastly, the ATCC 19606 genetic variant was isolated from the urinary tract [32,33,34]. The hypervirulent HUMC1 strain is a bacteremia isolate that is not directly associated with tissue and is in the dissemination phase of infection. This hypervirulent strain utilizes Zot to disassemble tight junctions (TJs) and disseminate through epithelial tissues without causing tissue damage. As mentioned earlier, Zot’s functions are reversible and are not tissue damaging. Not only is HUMC1 in the dissemination phase of infection, but it also most likely goes undetected in the circulatory system due to the lack of endotoxin shedding, strongly suggesting a new stealth-like mechanism that avoids the induction of sepsis. In stark contrast to hypervirulent HUMC1 isolates, the other strains tested employ tissue adhesion, biofilm formation, and antibiotic resistance as a mechanism of pathogenesis, whereas the hypervirulent HUMC1 displays dissemination characteristics very early on during bacteremia infection.

A retrospective study comparing the mortality rates between 252 patients infected with biofilm-forming *A. baumannii* bacteremia clinical strains vs. 459 patients infected with non-biofilm-forming *A. baumannii* bacteremia isolates showed that there was no significant difference between the 28-day survival and non-survival groups, in terms of the biofilm-forming ability, compared to the patients infected with non-biofilm-forming isolates. Those patients infected with biofilm-forming bacteremia strains had a lower in-hospital mortality rate [73]. This study implies that when *A. baumannii* is in a bacteremia phase of infection, it employs an alternative mechanism of pathogenesis irrespective of whether it can form biofilms. Our results show that HUMC1 cells have the capability to reach higher levels of quorum, compared to their counterparts, allowing them to produce high levels of Zot to disassemble TJs, thereby enabling dissemination through epithelial tissues and entry into or exit from the circulatory system. Additionally, the fact that HUMC1 cells do not shed endotoxin strongly supports our hypothesis that this hypervirulent strain is adopting stealth-like capabilities, whereby HUMC1 cells travel undetected during circulatory dissemination. For example, our model predicts that a ventilator-associated *A. baumannii* infection first establishes a local pneumonic infection by adhering to alveolar tissue and forming a biofilm. As biofilm formation progresses, *A. baumannii* begins to promote the accelerated decline in pulmonary function, followed by rapid paracellular transport between alveolar epithelial tissue, mediated by Zot. As *A. baumannii* reaches the circulatory system, it transitions to a bacteremia state, where it disseminates undetected, while secreting Zot, whose reversible functions do not seem to cause tissue damage.

There are many avenues of research to pursue pertaining to the data presented here. First, it would be of great interest to assess whether the increase in HUMC1 cell density is associated with quorum sensing (QS) capacities and whether Zot secretion is regulated by such a process. To date, the AbaI/AbaR system has been identified as a canonical QS mechanism in *A. baumannii* [41]. Knocking out such QS genes in HUMC1 cells will reveal their regulatory capabilities as it relates to Zot secretion at higher levels of cell density. Furthermore, the translation of our in vitro studies into in vivo models will enable further assessment as to how relevant Zot is in the pathogenesis of *A. baumannii*. Comparative in vivo studies using wildtype HUMC1 cells vs. HUMC1∆Zot gene knockouts will reveal its significance. Since HUMC1 cells display extended replication capacity, it is compelling to correlate higher bacterial burden in the organs of infected mice compared to in mice infected with the other clinical isolates. Furthermore, as our in vitro data show, HUMC1 cells do not shed endotoxin into the extracellular milieu, and it would be interesting to determine whether HUMC1-infected mice develop sepsis compared to the other counterparts that are strong endotoxin shedders. Such studies will further confirm our current hypothesis stating that the hypervirulent bacteremia strain HUMC1 adopts a stealth-like infection during dissemination in the circulatory system. Lastly, the most intriguing question remaining is how can HUMC1 and ATCC 17978 clinical isolates be so phenotypically different when their genomes share 98% identity. One can explore the mechanisms that trigger HUMC1 cells to transition from a bacteremia phase to passing the blood–brain-barrier with the help of Zot and establishing meningeal biofilms, as is the case with clinical isolate ATCC 17978 [49,74,75,76,77].

## 4. Materials and Methods

### 4.1. Strains

Six clinical isolates of *Acinetobacter baumannii* were used. Three of the strains (HUMC1, HUMC6, and HUMC12) were isolated from patients seen at Harbor-UCLA Medical Center. HUMC1 was isolated from the blood and sputum of the patient. HUMC6 was isolated from a patient with ventilation-associated pneumonia, and HUMC12 was isolated from a diabetic stump wound. *A. baumannii* ATCC 17978 was isolated in 1951 from a 4-month-old infant with fatal meningitis. Lastly, ATCC 19606 was isolated from a urinary tract infection in the early 1900s [32,33,34]. All strains were grown in three types of media: tryptic soy broth (TSB), DMEMF12 + 10% fetal bovine serum (FBS), and DMEMF12 + 10% TSB. (Gibco DMEMF12, without phenol red was supplied by Thermo Fisher Scientific, Los Angeles, CA, USA).

### 4.2. Antibiotic Resistance

The following antibiotics were chosen to assess susceptibility: kanamycin, neomycin, tetracycline, streptomycin, and chloramphenicol. All antibiotics were obtained from Sigma Chemical Company, St. Louis, MO, USA. Prior to adding antibiotics to the molten agar mix, all reagents were autoclaved (103 kPa, 121 °C, 15 min), and all aqueous solutions of antibiotics were filter-sterilized through a 2 μm filter and were aseptically diluted to the desired concentration. Neomycin was dissolved in phosphate buffer solution (pH 7.2); streptomycin, tetracycline, kanamycin, and chloramphenicol were all dissolved in water. Agar plates were prepared by dissolving 40 g of Difco TSB (Becton Dickinson, Franklin Lakes, NJ, USA) in 1 L of sterile water. The agar medium was agitated and heated to aid dissolution and then autoclaved (103 kPa, 121 °C, 15 min). After the agar medium had cooled to less than 45 °C, antibiotics were added to obtain a final concentration ranging between 10 and 350 μg mL^−1^, followed by pouring onto Petri dish plates. One set of control plates (containing no antibiotics) was prepared along with a set of plates for each of the five antibiotics used. Each strain was streaked across a Petri dish with varying antibiotic concentrations and incubated at 37 °C overnight.

### 4.3. Growth Kinetics

Growth curves of the bacterial isolates were assessed in TSB alone and DMEMF12 media supplemented with 10% TSB or 10% FBS (mimicking host cell environment). All experiments were performed in 96-well microtiter plates. All clinical isolates were cultivated at 37 °C (or 30–32 °C for longer incubation time/lengthier timepoints), with continuous shaking for 36 h. Cell density was assessed by spectrophotometry at OD 600 every thirty minutes. For quantitative purposes, we used eight replicate wells for each strain.

### 4.4. Endotoxin Shedding

The Pierce™ Chromogenic Endotoxin Quant Kit (cat. #A39552, Thermo Fisher Scientific, Los Angeles, CA, USA) was used to evaluate the concentration of endotoxin in the cell-free culture supernatants. All procedures were performed in accordance with the manufacturer’s protocol. Briefly, *E. coli* endotoxin was used as a standard to measure endotoxin concentration in different supernatants. First, 50 μL of control samples, endotoxin standards, and cell-free culture supernatants was added to each well of a 96-well plate. Second, 50 μL of amoebocyte lysis reagent was added, and the plate was incubated for 10 min at 37 °C. Following incubation, an equal volume of pre-warmed chromogenic substrate solution was added to the plate and incubated at 37 °C for an additional 15 min. Lastly, 25% acetic acid was added as stop solution. Colorimetric differences were quantified using a microplate reader set to OD450 nm.

### 4.5. Biofilm Quantification Assays

To assess biofilm production capabilities, quantitative biofilm assays were performed under different types of media conditions (100% TSB, DMEMF12 + 10% TSB, DMEMF12 + 10% FBS). All strains were grown overnight in a rich medium (i.e., TSB). After overnight culture, samples were diluted 1:100 into fresh TSB medium and sub-cultured for an additional three hours, at which point, cells were quantified using a spectrophotometer at OD600. Approximately 10^7^ cells of culture were added to each well in a 96-well plate. For quantitative purposes, we used eight replicate wells for each strain. After the addition of cells, the microtiter plate was incubated for 24–48 h at 37 °C. After incubation, cells were dumped out by turning the plate over and shaking out the liquid. Approximately 100 μL of PBS was then added to each well to rinse any residual unattached cells and media components that can be stained in the next step. This process was repeated three times. XTT detection solution was used to quantify biofilm formation in each well as OD450.

### 4.6. Heat Inactivation Experiments

HUMC1 supernatants were heat-inactivated by incubating samples in a boiling water bath for 26 min, followed by immediate incubation on ice. Supernatant samples were then sterilized with a 0.2 μm filter, and sterile samples were added to A549 cells seeded onto a 96-well plate, followed by overnight incubation at 37 °C with 5% CO_2_. Microscopy was performed the next day to assess cell morphology.

### 4.7. Supernatant Fractionations and Microscopy

To assess the molecular weight range of the exotoxin, we used the human alveolar epithelial cell line (A549) incubated with HUMC1 cell supernatants. Using a spectrophotometer set to OD600, all strains were first normalized to OD 0.5 (~2 × 10^8^ cells/mL) and then cultured in DMEM/F12 media, without phenol red, supplemented with 10% FBS, for approximately 48 h at 37 °C with shaking. After this, cell-free supernatants were obtained by centrifugation, followed by filtration through a 0.2 µm membrane filter. Then, 100 µL of unfractionated or fractioned (by employing size exclusion chromatography using membrane cut-off columns of 50, 30, 10, 5 kDa) cell-free culture supernatants were added onto confluent A549 alveolar epithelial cells seeded in a 96-well plate and incubated overnight at 37 °C with 5% CO_2_. Microscopy was performed the next day to assess cell morphology.

### 4.8. UniProt Database Mining

The UniProt provides a high-quality and comprehensive set of protein sequences annotated with functional information. To fully understand the distribution of the Zot gene among *A. baumannii*, we performed an extensive UniProt search with Zonula occludens toxin as the query (accessed on 26 February 2024). Of the 284 predicted individual protein entities identified, the closest full-length matches (≥345 amino acids) to the *Vibrio cholerae* Zot protein sequence were chosen. A total of 35 different protein entities and 6 *A. baumannii*-specific bacteriophages were identified and chosen for our studies (Figure 7). All the identified protein entities were specific for *A. baumannii* [31,78,79].

### 4.9. NCBI BLAST and MUSCLE Algorithm

Initially, a phylogenetic tree was developed by employing the MUSCLE algorithm, which counts the number of short sub-sequences (known as k-mers, k-tuples, or words) that two sequences have in common, without constructing an alignment. In the second step, MUSCLE uses the tree to construct a progressive alignment. At each node of the binary tree, a pair-wise alignment is constructed, progressing from leaves towards the root. The first alignment is made from two sequences. Later alignments included one of three different types: sequence–sequence, profile–sequence, or profile–profile, where “profile” means the multiple alignment of the sequences under a given internal node of the tree. In conjunction with MUSCLE, we employed the Multiple Sequence Alignment Viewer Application (MSA), which is a web-based application that displays the graphical alignments generated by algorithms such as CLUSTALW and MUSCLE and alignments from NCBI BLAST. We viewed the alignments using the FASTA files generated by MUSCLE, UniProt, and NCBI [31,80,81,82].

### 4.10. Backtranslation

To compare the amino acid sequence homology among all the tested *A. baumannii*-specific Zot protein sequences, a backtranslation approach was used for HUMC1, ATCC 17978, and ATCC 19606 and compared to the rest of the strains identified in UniProt. Backtranslation is a method of decoding amino acid sequences into their corresponding codons. However, genome degeneracy makes backtranslation very ambiguous, since multiple codons can represent the same amino acid. The most common strategy to overcome such a problem is based on the imitation of codon usage within the target species. Accordingly, all the Zot protein sequences were backtranslated according to *A. baumannii*-specific codon optimization tools such as EMBOSS Backtranseq [31,83,84].

### 4.11. SWISS-MODEL

SWISS-MODEL is an algorithm used for the automated comparative modeling of protein structures. In the “first approach mode”, only an amino acid sequence of a protein is submitted to build a 3D model. Template selection, alignment, and model building are completely automated by the server. In the “alignment mode”, the modeling process is based on a user-defined target–template alignment. The user specifies which sequence in the given alignment is the target sequence and which one corresponds to a structurally known protein chain from the ExPDB template library. The server will build the model based on the given alignment. Complex modeling tasks can be handled via the “project mode” using DeepView (Swiss-PdbViewer), an integrated sequence-to-structure workbench [31,85,86].

## Figures and Tables

**Table 1:** Hypervirulent HUMC1 cells are not endotoxin shedders. Endotoxin levels were quantified in different types of cell-free culture supernatant fractions derived from five different *A. baumannii* strains. The kit used for measurement employed a standard curve from 0 to 1.0 Eu/Ml, and supernatants from HUMC1 were measured undiluted. In contrast, the supernatants from the other four strains were diluted 1:1000. Note the lack of endotoxin shedding in the HUMC1 clinical isolate. Endotoxin concentration was measured in Eu/mL.

Strain	HUMC1	HUMC6	HUMC12	ATCC 17978	ATCC 19606
TSB	0.32	3.7 × 10^3^	3.6 × 10^3^	3.7 × 10^3^	3.5 × 10^3^
DMEMF12 + 10% TSB	0.65	1.5 × 10^3^	1.6 × 10^3^	1.6 × 10^3^	1.6 × 10^3^
DMEMF12 + 10% FBS	0.34	1.8 × 10^3^	1.7 × 10^3^	1.6 × 10^3^	1.8 × 10^3^

## Data Availability

The original contributions presented in this study are included in this article; further inquiries can be directed to the author.
